# (6aS*,6bS*,11R*,11aR*)-6-(2-Furyl­methyl)-5,12-dioxo-5,6,6a,6b,7,11,11a,12-octa­hydro­furo[3′,2′:5,6]isoindolo[2,1-*a*]quinazoline-11-carb­oxy­lic acid

**DOI:** 10.1107/S160053681104311X

**Published:** 2011-10-29

**Authors:** Mykola D. Obushak, Yuri I. Horak, Vladimir P. Zaytsev, Ekaterina L. Motorygina, Fedor I. Zubkov, Victor N. Khrustalev

**Affiliations:** a Department of Organic Chemistry, Ivan Franko National University of Lviv, 6 Kyryla i Mefodiya Street, Lviv 79005, Ukraine; b Organic Chemistry Department, Russian People’s Friendship University, Miklukho-Maklaya Street 6, Moscow, 117198, Russian Federation; c X-ray Structural Centre, A. N. Nesmeyanov Institute of Organoelement Compounds, Russian Academy of Sciences, 28 Vavilov Street, B-334, Moscow 119991, Russian Federation

## Abstract

The title compound, C_23_H_18_N_2_O_6_, is the product of an intra­molecular thermal cyclo­addition within 1-malein-2-[(*E*)-2-(2-fur­yl)vin­yl]-4-oxo-3,4-dihydro­quinazoline. The mol­ecule comprises a previously unknown fused penta­cyclic system containing two five-membered rings (2-pyrrolidinone and furan) and three six-membered rings (benzene, 2,3-dihydro-4-pyrimidinone and dihydro­cyclo­hexa­ne). The central five-membered pyrrolidinone ring has the usual envelope conformation. The six-membered dihydro­pyrimidinone and dihydro­cyclo­hexane rings adopt a half-boat and a half-chair conformation, respectively. The dihedral angle between the planes of the terminal benzene and furan rings is 45.99 (7)°. In the crystal, O—H⋯O hydrogen bonds link the mol­ecules into centrosymmetric dimers. Weak C—H⋯O hydrogen bonds consolidate further the crystal packing, which exhibits π–π inter­actions, with a short distance of 3.556 (3) Å between the centroids of benzene rings of neighbouring mol­ecules.

## Related literature

For 2-vinyl­furans as dienes, see: Kotsuki *et al.* (1981 ▶); Keil *et al.* (1990 ▶); Kusurkar & Bhosale (1990 ▶); Anisimova *et al.* (2006 ▶). For the intra­molecular Diels–Alder reaction for furan (IMDAF), see: Vogel *et al.* (1999 ▶); Zubkov *et al.* (2005 ▶, 2009 ▶, 2010 ▶). For related compounds, see: Chou & Tsai (1992 ▶); Chou *et al.* (1997 ▶); Sun & Murray (1999 ▶); Ohno *et al.* (2005 ▶); Patre *et al.* (2007 ▶).
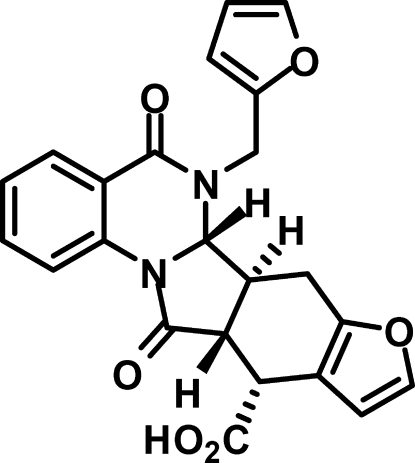


## Experimental

### 

#### Crystal data

C_23_H_18_N_2_O_6_
*M* *_r_* = 418.39
Monoclinic, 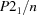

*a* = 8.2364 (5) Å
*b* = 16.9882 (10) Å
*c* = 13.1568 (8) Å
β = 99.102 (1)°
*V* = 1817.74 (19) Å^3^
*Z* = 4
Mo *K*α radiation
μ = 0.11 mm^−1^
*T* = 100 K
0.30 × 0.20 × 0.18 mm

#### Data collection

Bruker SMART 1K CCD diffractometer
Absorption correction: multi-scan (*SADABS*; Sheldrick, 1998 ▶) *T*_min_ = 0.967, *T*_max_ = 0.980
21001 measured reflections
5293 independent reflections
4139 reflections with *I* > 2σ(*I*)
*R*_int_ = 0.027

#### Refinement

*R*[*F* ^2^ > 2σ(*F* ^2^)] = 0.060
*wR*(*F* ^2^) = 0.160
*S* = 1.00
5293 reflections
280 parameters
H-atom parameters constrained
Δρ_max_ = 0.45 e Å^−3^
Δρ_min_ = −0.34 e Å^−3^

### 

Data collection: *SMART* (Bruker, 1998 ▶); cell refinement: *SAINT* (Bruker, 1998 ▶); data reduction: *SAINT*; program(s) used to solve structure: *SHELXTL* (Sheldrick, 2008 ▶); program(s) used to refine structure: *SHELXTL*; molecular graphics: *SHELXTL*; software used to prepare material for publication: *SHELXTL*.

## Supplementary Material

Crystal structure: contains datablock(s) global, I. DOI: 10.1107/S160053681104311X/cv5175sup1.cif

Structure factors: contains datablock(s) I. DOI: 10.1107/S160053681104311X/cv5175Isup2.hkl

Supplementary material file. DOI: 10.1107/S160053681104311X/cv5175Isup3.cml

Additional supplementary materials:  crystallographic information; 3D view; checkCIF report

## Figures and Tables

**Table 1:** Hydrogen-bond geometry (Å, °)

*D*—H⋯*A*	*D*—H	H⋯*A*	*D*⋯*A*	*D*—H⋯*A*
O4—H4O⋯O1^i^	0.93	1.75	2.671 (2)	174
C2—H2⋯O3^ii^	0.95	2.42	3.326 (2)	160
C3—H3⋯O3^iii^	0.95	2.56	3.384 (2)	146
C7—H7*B*⋯O4^iv^	0.99	2.54	3.455 (2)	155
C11*A*—H11*A*⋯O1^v^	1.00	2.38	3.325 (2)	157

Symmetry codes: (i) 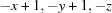; (ii) 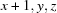; (iii) 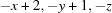; (iv) 
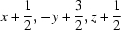
; (v) 
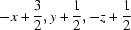
.

**Fig. 1.**
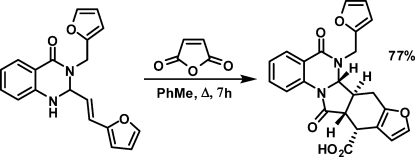
Reaction of 2-[(E)-2-(2-furyl)vinyl]-2,3-dihydroquinazolin-4-one with maleic anhydride.

**Fig. 2.**
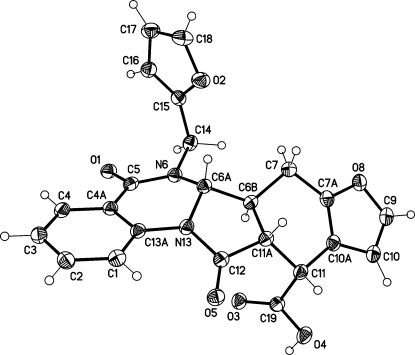
Molecular structure of (I). Displacement ellipsoids are shown at the 50% probability level.

## supplementary crystallographic information

### Comment

Currently, there are only a few reports concerning the [4 + 2] cycloaddition of 2-vinylfurans with dienophiles (Kotsuki *et al.*, 1981; Keil *et al.*, 1990; Kusurkar & Bhosale, 1990; Anisimova *et al.*, 2006). In the present work, within the scope of our investigations on the intramolecular Diels-Alder reaction of furan (IMDAF) (Vogel *et al.*, 1999; Zubkov *et al.*, 2005, 2009, 2010), we demonstrate the possibility of intramolecular thermal cycloaddition within 1-malein-2-[(*E*)-2-(2-furyl)vinyl]-4-oxo-3,4-dihydroquinazoline. The latter is an intermediate of a reaction of 2-[(*E*)-2-(2-furyl)vinyl]-2,3-dihydroquinazolin-4-one with maleic anhydride (Figure 1). The reaction product contains a previously unknown pentacycle bearing four asymmetrical centers. The main structural fragments of the new pentacycle are quinazoline and furo[2,3-*f*]isoindole (Chou & Tsai, 1992; Chou *et al.*, 1997; Sun & Murray, 1999; Ohno *et al.*, 2005; Patre *et al.*, 2007). The structure of the final product - 6-(2-furylmethyl)-5,12-dioxo-5,6,6a,6 b,7,11,11*a*,12-octahydrofuro[3',2':5,6]isoindolo[2,1-*a*]quinazoline-11-carboxylic acid, C_23_H_18_N_2_O_6_, (I) was unambiguously established by X-ray diffraction study.

Molecule of (I) comprises a fused pentacyclic system containing two five-membered rings (2-pyrrolidinone and furan) and three six-membered rings (benzene, 2,3-dihydro-4-pyrimidinone and dihydrocyclohexane) (Figure 2). The central five-membered pyrrolidinone ring has usual *envelope* conformation (the C6B carbon atom is out of the plane through the other atoms of the ring by 0.477 (2) Å)), and the central six-membered dihydropyrimidinone and dihydrocyclohexane rings adopt the nonsymmetrical *half-boat* (the N6 nitrogen and C6A carbon atoms are out of the plane through the other atoms of the ring by 0.265 (3) and 0.626 (3) Å, respectively) and nonsymmetrical *half-chair* (the C6B and C11A carbon atoms are out of the plane through the other atoms of the ring by -0.555 (3) and 0.281 (3) Å, respectively) conformations, respectively. The dihedral angle between the planes of the end-cutting benzene and furan rings is 45.99 (7)°.

The furylmethyl ligand and carboxylic acid substituent at the C11 atom arrange from different sides of the main pentacyclic framework. Apparently, such disposition is explained by the fact that, in the crystal, the molecules of (I) form the centrosymmerical dimers through the intermolecular O4—H4O···O1^i^ hydrogen bonding interactions (Table 1). Furthermore, due to the steric reasons within the dimers, the nitrogen N6 atom adopts a trigonal-pyramidal geometry (sum of the bond angles is 357.5°), while the nitrogen N13 atom has a trigonal-planar geometry (sum of the bond angles is 360.0°). Weak intermolecular C—H···O hydrogen bonds consolidate further the crystal packing, which exhibits π—π interactions with the short distance of 3.556 (3) Å between the centroids of benzene rings from the neighbouring molecules [Cg···Cg^i^; symmetry code (i) 1 - *x*, 1 - *y*, -*z*].

The molecule of (I) possesses four asymmetric centers at the C6A, C6B, C11 and C11A carbon atoms and can have potentially numerous diastereomers. The crystal of (I) is racemic and consists of enantiomeric pairs with the following relative configuration of the centers: *rac*-6a*S**,6 b*S**,11*R**,11*aR**.

### Experimental

A mixture of the initial 3-(2-furylmethyl)-2-[(*E*)-2-(2-furyl)vinyl]-2,3-dihydroquinazolin-4(1*H*)-one (0.5 g, 1.6 mmol) and maleic anhydride (0.17 g, 1.7 mmol) was refluxed for 8 h in toluene (10 ml). At the end of the reaction the resulting mixture was cooled, and formed brown precipitate was filtered off, washed with benzene(2x10 ml) and ether (2x10 ml). Further crystallization from an ethanol-DMF mixture gives the corresponding acid (0.5 g, 1.2 mmol) as orange prism. Yield is 77%. The single-crystal of the product was obtained by slow crystallization from an ethanol-ethyl acetate mixture. *M*.p. = 499–500 K. IR (KBr), ν/cm^-1^: 1630, 1727 (NCO, CO_2_H). ^1^H NMR (600 MHz, DMSO-*d*_6_, 293 K): δ = 12.7 (br.s,1*H*, CO_2_*H*), 8.24 (d, 1H,H4, J_4,3_ = 7.8), 7.93 (d, 1H, H1, J_1,2_ = 7.8), 7.60 (t, 1H, H2, J_1,2_ = J_2,3_ = 7.8), 7.57 (dd, 1H, H5', J_4',5'_= 1.8, J_3',5'_ = 0.8), 7.54 (d, 1H, H9, J_9,10_ = 0.9), 7.25 (t, 1H, H3, J_2,3_ = J_3,4_ = 7.8), 6.44 (d, 1H, H10, J_9,10_= 0.9), 6.38 (dd, 1H, H4', J_3',4'_ = 3.2, J_4',5'_ = 1.8), 6.32 (d, 1H, H3', J_3',4'_ = 3.2), 5.53 (d, 1H, H6A, J_6 A,6B_ = 8.2), 5.01 (d, 1H, NCH_2_, J_H14A,H14B_ = 16.9), 4.73 (d,1*H*, NCH_2_, J_H14A,H14B_ = 16.9), 3.83 (d, 1H, H_11_, J_11,11 A_ = 5.0), 3.29 (m, 1H, H6B), 3.16 (dd, 1H, H7A, J_7 A,7B_ = 15.6, J_7 A,6B_ = 4.6), 3.04 (dd, 1H, H11A, J_11 A,6B_ = 12.4, J_11,11 A_ = 5.0), 2.70 (dd, 1H, H7B, J_7 A,7B_ = 15.6, J_7B,6B_ = 11.5). Mass spectrum (EI—MS, 70 eV) *m/z*(I_r_, (%)): 418 [*M*^+^] (100), 374 (22), 322 (18), 276 (41), 236 (13), 227 (16), 185 (12), 147 (13), 119 (14), 91 (37), 81 (75), 53 (12). Anal. Calcd. for C_23_H_18_N_2_O_6_: C, 66.02; H, 4.34; N, 6.70. Found: C, 66.12; H, 4.04; N, 6.83.

### Refinement

The hydroxyl hydrogen atom was localized in the difference-Fourier map and included in the refinement with fixed positional and isotropic displacement parameters [*U*_iso_(H) = 1.5*U*_eq_(O)]. The other hydrogen atoms were placed in calculated positions with C–H = 0.95–1.00 Å and refined in the riding model with fixed isotropic displacement parameters [*U*_iso_(H) = 1.2*U*_eq_(C)].

### Crystal data

**Table:**

C_23_H_18_N_2_O_6_	*F*(000) = 872
*M**_r_* = 418.39	*D*_x_ = 1.529 Mg m^−^^3^
Monoclinic, *P*2_1_/*n*	Mo *K*α radiation, λ = 0.71073 Å
Hall symbol: -P 2yn	Cell parameters from 6904 reflections
*a* = 8.2364 (5) Å	θ = 2.4–30.0°
*b* = 16.9882 (10) Å	µ = 0.11 mm^−^^1^
*c* = 13.1568 (8) Å	*T* = 100 K
β = 99.102 (1)°	Prism, orange
*V* = 1817.74 (19) Å^3^	0.30 × 0.20 × 0.18 mm
*Z* = 4	

### Data collection

**Table:**

Bruker SMART 1K CCD diffractometer	5293 independent reflections
Radiation source: fine-focus sealed tube	4139 reflections with *I* > 2σ(*I*)
graphite	*R*_int_ = 0.027
φ and ω scans	θ_max_ = 30.0°, θ_min_ = 2.0°
Absorption correction: multi-scan (*SADABS*; Sheldrick, 1998)	*h* = −11→11
*T*_min_ = 0.967, *T*_max_ = 0.980	*k* = −23→23
21001 measured reflections	*l* = −18→18

### Refinement

**Table:**

Refinement on *F*^2^	Primary atom site location: structure-invariant direct methods
Least-squares matrix: full	Secondary atom site location: difference Fourier map
*R*[*F*^2^ > 2σ(*F*^2^)] = 0.060	Hydrogen site location: difference Fourier map
*wR*(*F*^2^) = 0.160	H-atom parameters constrained
*S* = 1.00	*w* = 1/[σ^2^(*F*_o_^2^) + (0.08*P*)^2^ + 1.9*P*] where *P* = (*F*_o_^2^ + 2*F*_c_^2^)/3
5293 reflections	(Δ/σ)_max_ < 0.001
280 parameters	Δρ_max_ = 0.45 e Å^−^^3^
0 restraints	Δρ_min_ = −0.34 e Å^−^^3^

### Special details

**Table:**

Geometry. All e.s.d.'s (except the e.s.d. in the dihedral angle between two l.s. planes) are estimated using the full covariance matrix. The cell e.s.d.'s are taken into account individually in the estimation of e.s.d.'s in distances, angles and torsion angles; correlations between e.s.d.'s in cell parameters are only used when they are defined by crystal symmetry. An approximate (isotropic) treatment of cell e.s.d.'s is used for estimating e.s.d.'s involving l.s. planes.
Refinement. Refinement of *F*^2^ against ALL reflections. The weighted *R*-factor *wR* and goodness of fit *S* are based on *F*^2^, conventional *R*-factors *R* are based on *F*, with *F* set to zero for negative *F*^2^. The threshold expression of *F*^2^ > σ(*F*^2^) is used only for calculating *R*-factors(gt) *etc*. and is not relevant to the choice of reflections for refinement. *R*-factors based on *F*^2^ are statistically about twice as large as those based on *F*, and *R*- factors based on ALL data will be even larger.

### Fractional atomic coordinates and isotropic or equivalent isotropic displacement parameters (Å^2^)

**Table:**

	*x*	*y*	*z*	*U*_iso_*/*U*_eq_	
O1	0.77116 (15)	0.37826 (7)	0.24161 (9)	0.0235 (3)	
O2	0.73217 (17)	0.53678 (7)	0.48118 (10)	0.0286 (3)	
O3	0.47571 (15)	0.62107 (7)	−0.02374 (9)	0.0245 (3)	
O4	0.31904 (17)	0.72270 (7)	−0.08729 (10)	0.0284 (3)	
H4O	0.2827	0.6861	−0.1383	0.043*	
O5	0.82503 (15)	0.69632 (7)	0.03174 (9)	0.0237 (3)	
C1	1.0826 (2)	0.57749 (10)	0.10046 (13)	0.0221 (3)	
H1	1.0901	0.6300	0.0769	0.027*	
C2	1.2040 (2)	0.52240 (10)	0.08887 (13)	0.0237 (3)	
H2	1.2942	0.5378	0.0565	0.028*	
C3	1.1958 (2)	0.44550 (10)	0.12353 (13)	0.0248 (3)	
H3	1.2811	0.4092	0.1165	0.030*	
C4	1.0627 (2)	0.42198 (10)	0.16832 (13)	0.0231 (3)	
H4	1.0557	0.3693	0.1914	0.028*	
C4A	0.93842 (19)	0.47584 (9)	0.17967 (12)	0.0197 (3)	
C5	0.7966 (2)	0.44955 (9)	0.22637 (12)	0.0203 (3)	
N6	0.69629 (17)	0.50537 (8)	0.25528 (10)	0.0198 (3)	
C6A	0.72967 (19)	0.59004 (9)	0.24741 (12)	0.0188 (3)	
H6A	0.7925	0.6104	0.3135	0.023*	
C6B	0.57091 (19)	0.63798 (9)	0.21574 (12)	0.0192 (3)	
H6B	0.4918	0.6042	0.1694	0.023*	
C7	0.4786 (2)	0.67373 (10)	0.29657 (13)	0.0224 (3)	
H7A	0.4284	0.6322	0.3343	0.027*	
H7B	0.5529	0.7060	0.3465	0.027*	
C7A	0.3497 (2)	0.72356 (9)	0.23479 (13)	0.0220 (3)	
O8	0.20912 (15)	0.74143 (7)	0.27305 (10)	0.0246 (3)	
C9	0.1130 (2)	0.78350 (10)	0.19758 (14)	0.0257 (3)	
H9	0.0071	0.8036	0.2030	0.031*	
C10	0.1885 (2)	0.79273 (10)	0.11445 (14)	0.0240 (3)	
H10	0.1471	0.8199	0.0526	0.029*	
C10A	0.3445 (2)	0.75302 (9)	0.13854 (13)	0.0208 (3)	
C11	0.4827 (2)	0.74087 (9)	0.07672 (12)	0.0202 (3)	
H11	0.5173	0.7930	0.0522	0.024*	
C11A	0.62601 (19)	0.70466 (9)	0.15030 (12)	0.0189 (3)	
H11A	0.6735	0.7472	0.1986	0.023*	
C12	0.76530 (19)	0.67005 (9)	0.10328 (12)	0.0196 (3)	
N13	0.82043 (16)	0.60449 (8)	0.16182 (10)	0.0196 (3)	
C13A	0.95024 (19)	0.55390 (9)	0.14728 (12)	0.0193 (3)	
C14	0.5826 (2)	0.48033 (9)	0.32540 (13)	0.0220 (3)	
H14A	0.5286	0.4304	0.3003	0.026*	
H14B	0.4961	0.5207	0.3261	0.026*	
C15	0.6729 (2)	0.46908 (10)	0.43133 (13)	0.0220 (3)	
C16	0.7223 (2)	0.40551 (10)	0.48999 (13)	0.0244 (3)	
H16	0.6980	0.3520	0.4731	0.029*	
C17	0.8185 (2)	0.43469 (12)	0.58253 (14)	0.0290 (4)	
H17	0.8700	0.4044	0.6393	0.035*	
C18	0.8216 (3)	0.51364 (12)	0.57333 (14)	0.0325 (4)	
H18	0.8777	0.5484	0.6236	0.039*	
C19	0.4283 (2)	0.68777 (10)	−0.01577 (12)	0.0212 (3)	

### Atomic displacement parameters (Å^2^)

**Table:**

	*U*^11^	*U*^22^	*U*^33^	*U*^12^	*U*^13^	*U*^23^
O1	0.0327 (6)	0.0167 (5)	0.0209 (6)	−0.0006 (4)	0.0031 (5)	0.0008 (4)
O2	0.0387 (7)	0.0233 (6)	0.0229 (6)	−0.0022 (5)	0.0021 (5)	−0.0022 (5)
O3	0.0280 (6)	0.0195 (6)	0.0251 (6)	0.0020 (4)	0.0017 (5)	−0.0018 (4)
O4	0.0352 (7)	0.0225 (6)	0.0241 (6)	0.0061 (5)	−0.0060 (5)	−0.0028 (5)
O5	0.0253 (6)	0.0211 (6)	0.0250 (6)	−0.0016 (4)	0.0055 (5)	0.0028 (4)
C1	0.0214 (7)	0.0221 (7)	0.0222 (7)	−0.0022 (6)	0.0015 (6)	−0.0004 (6)
C2	0.0204 (7)	0.0285 (8)	0.0217 (8)	−0.0005 (6)	0.0017 (6)	−0.0034 (6)
C3	0.0248 (8)	0.0270 (8)	0.0217 (7)	0.0044 (6)	0.0011 (6)	−0.0023 (6)
C4	0.0289 (8)	0.0198 (7)	0.0199 (7)	0.0044 (6)	0.0014 (6)	0.0005 (6)
C4A	0.0232 (7)	0.0184 (7)	0.0169 (7)	0.0007 (5)	0.0017 (5)	−0.0006 (5)
C5	0.0242 (7)	0.0182 (7)	0.0174 (7)	0.0001 (5)	−0.0006 (6)	0.0011 (5)
N6	0.0230 (6)	0.0166 (6)	0.0198 (6)	−0.0007 (5)	0.0032 (5)	0.0011 (5)
C6A	0.0212 (7)	0.0160 (7)	0.0188 (7)	−0.0010 (5)	0.0016 (5)	0.0008 (5)
C6B	0.0203 (7)	0.0182 (7)	0.0188 (7)	0.0005 (5)	0.0017 (5)	0.0022 (5)
C7	0.0239 (7)	0.0225 (7)	0.0212 (7)	0.0011 (6)	0.0044 (6)	0.0011 (6)
C7A	0.0221 (7)	0.0187 (7)	0.0251 (8)	0.0000 (5)	0.0037 (6)	−0.0031 (6)
O8	0.0239 (6)	0.0223 (6)	0.0284 (6)	0.0025 (4)	0.0066 (5)	−0.0004 (5)
C9	0.0243 (8)	0.0202 (7)	0.0321 (9)	0.0026 (6)	0.0030 (6)	−0.0017 (6)
C10	0.0237 (7)	0.0196 (7)	0.0279 (8)	0.0020 (6)	0.0009 (6)	−0.0016 (6)
C10A	0.0225 (7)	0.0159 (7)	0.0235 (8)	−0.0001 (5)	0.0019 (6)	−0.0020 (5)
C11	0.0224 (7)	0.0166 (7)	0.0210 (7)	0.0006 (5)	0.0017 (6)	0.0007 (5)
C11A	0.0201 (7)	0.0165 (6)	0.0195 (7)	−0.0015 (5)	0.0010 (5)	0.0002 (5)
C12	0.0213 (7)	0.0163 (6)	0.0203 (7)	−0.0021 (5)	0.0011 (5)	−0.0009 (5)
N13	0.0200 (6)	0.0172 (6)	0.0216 (6)	0.0008 (5)	0.0034 (5)	0.0019 (5)
C13A	0.0192 (7)	0.0198 (7)	0.0181 (7)	0.0007 (5)	0.0002 (5)	0.0001 (5)
C14	0.0211 (7)	0.0189 (7)	0.0264 (8)	−0.0011 (5)	0.0044 (6)	0.0032 (6)
C15	0.0240 (7)	0.0207 (7)	0.0221 (7)	−0.0005 (6)	0.0056 (6)	−0.0005 (6)
C16	0.0243 (8)	0.0243 (8)	0.0252 (8)	0.0001 (6)	0.0058 (6)	0.0032 (6)
C17	0.0288 (8)	0.0358 (9)	0.0229 (8)	0.0001 (7)	0.0051 (7)	0.0046 (7)
C18	0.0394 (10)	0.0372 (10)	0.0201 (8)	−0.0038 (8)	0.0019 (7)	−0.0021 (7)
C19	0.0218 (7)	0.0207 (7)	0.0209 (7)	0.0002 (6)	0.0026 (6)	0.0008 (6)

### Geometric parameters (Å, °)

**Table:**

O1—C5	1.2507 (19)	C7—C7A	1.494 (2)
O2—C18	1.373 (2)	C7—H7A	0.9900
O2—C15	1.375 (2)	C7—H7B	0.9900
O3—C19	1.209 (2)	C7A—C10A	1.356 (2)
O4—C19	1.334 (2)	C7A—O8	1.368 (2)
O4—H4O	0.9287	O8—C9	1.369 (2)
O5—C12	1.214 (2)	C9—C10	1.350 (3)
C1—C13A	1.393 (2)	C9—H9	0.9500
C1—C2	1.395 (2)	C10—C10A	1.442 (2)
C1—H1	0.9500	C10—H10	0.9500
C2—C3	1.389 (2)	C10A—C11	1.514 (2)
C2—H2	0.9500	C11—C19	1.524 (2)
C3—C4	1.383 (2)	C11—C11A	1.532 (2)
C3—H3	0.9500	C11—H11	1.0000
C4—C4A	1.398 (2)	C11A—C12	1.506 (2)
C4—H4	0.9500	C11A—H11A	1.0000
C4A—C13A	1.401 (2)	C12—N13	1.389 (2)
C4A—C5	1.473 (2)	N13—C13A	1.408 (2)
C5—N6	1.351 (2)	C14—C15	1.485 (2)
N6—C6A	1.471 (2)	C14—H14A	0.9900
N6—C14	1.477 (2)	C14—H14B	0.9900
C6A—N13	1.467 (2)	C15—C16	1.352 (2)
C6A—C6B	1.541 (2)	C16—C17	1.432 (3)
C6A—H6A	1.0000	C16—H16	0.9500
C6B—C7	1.527 (2)	C17—C18	1.347 (3)
C6B—C11A	1.534 (2)	C17—H17	0.9500
C6B—H6B	1.0000	C18—H18	0.9500
			
C18—O2—C15	106.38 (14)	C9—C10—C10A	106.03 (15)
C19—O4—H4O	108.4	C9—C10—H10	127.0
C13A—C1—C2	118.66 (15)	C10A—C10—H10	127.0
C13A—C1—H1	120.7	C7A—C10A—C10	105.85 (15)
C2—C1—H1	120.7	C7A—C10A—C11	122.43 (15)
C3—C2—C1	121.48 (16)	C10—C10A—C11	131.71 (15)
C3—C2—H2	119.3	C10A—C11—C19	111.01 (13)
C1—C2—H2	119.3	C10A—C11—C11A	106.54 (13)
C4—C3—C2	119.64 (15)	C19—C11—C11A	111.52 (13)
C4—C3—H3	120.2	C10A—C11—H11	109.2
C2—C3—H3	120.2	C19—C11—H11	109.2
C3—C4—C4A	119.95 (15)	C11A—C11—H11	109.2
C3—C4—H4	120.0	C12—C11A—C11	117.25 (13)
C4A—C4—H4	120.0	C12—C11A—C6B	104.73 (12)
C4—C4A—C13A	119.98 (15)	C11—C11A—C6B	112.60 (13)
C4—C4A—C5	119.23 (14)	C12—C11A—H11A	107.3
C13A—C4A—C5	120.79 (14)	C11—C11A—H11A	107.3
O1—C5—N6	120.60 (15)	C6B—C11A—H11A	107.3
O1—C5—C4A	121.65 (15)	O5—C12—N13	126.00 (15)
N6—C5—C4A	117.73 (14)	O5—C12—C11A	127.11 (14)
C5—N6—C6A	122.48 (14)	N13—C12—C11A	106.78 (13)
C5—N6—C14	116.74 (13)	C12—N13—C13A	127.06 (14)
C6A—N6—C14	118.00 (13)	C12—N13—C6A	113.46 (13)
N13—C6A—N6	109.94 (12)	C13A—N13—C6A	119.47 (13)
N13—C6A—C6B	102.57 (12)	C1—C13A—C4A	120.24 (15)
N6—C6A—C6B	112.07 (13)	C1—C13A—N13	123.27 (14)
N13—C6A—H6A	110.7	C4A—C13A—N13	116.46 (14)
N6—C6A—H6A	110.7	N6—C14—C15	110.51 (13)
C6B—C6A—H6A	110.7	N6—C14—H14A	109.5
C7—C6B—C11A	108.73 (13)	C15—C14—H14A	109.5
C7—C6B—C6A	121.09 (13)	N6—C14—H14B	109.5
C11A—C6B—C6A	103.19 (12)	C15—C14—H14B	109.5
C7—C6B—H6B	107.7	H14A—C14—H14B	108.1
C11A—C6B—H6B	107.7	C16—C15—O2	110.11 (15)
C6A—C6B—H6B	107.7	C16—C15—C14	134.38 (16)
C7A—C7—C6B	103.64 (13)	O2—C15—C14	115.31 (14)
C7A—C7—H7A	111.0	C15—C16—C17	106.55 (16)
C6B—C7—H7A	111.0	C15—C16—H16	126.7
C7A—C7—H7B	111.0	C17—C16—H16	126.7
C6B—C7—H7B	111.0	C18—C17—C16	106.50 (16)
H7A—C7—H7B	109.0	C18—C17—H17	126.8
C10A—C7A—O8	111.01 (15)	C16—C17—H17	126.8
C10A—C7A—C7	129.18 (15)	C17—C18—O2	110.46 (16)
O8—C7A—C7	119.72 (15)	C17—C18—H18	124.8
C7A—O8—C9	105.95 (13)	O2—C18—H18	124.8
C10—C9—O8	111.16 (15)	O3—C19—O4	123.18 (15)
C10—C9—H9	124.4	O3—C19—C11	124.47 (15)
O8—C9—H9	124.4	O4—C19—C11	112.35 (14)
			
C13A—C1—C2—C3	−0.5 (2)	C19—C11—C11A—C6B	−76.15 (17)
C1—C2—C3—C4	1.6 (3)	C7—C6B—C11A—C12	158.92 (12)
C2—C3—C4—C4A	−0.8 (2)	C6A—C6B—C11A—C12	29.17 (15)
C3—C4—C4A—C13A	−1.1 (2)	C7—C6B—C11A—C11	−72.59 (16)
C3—C4—C4A—C5	179.25 (15)	C6A—C6B—C11A—C11	157.66 (13)
C4—C4A—C5—O1	−11.2 (2)	C11—C11A—C12—O5	39.4 (2)
C13A—C4A—C5—O1	169.16 (15)	C6B—C11A—C12—O5	165.06 (16)
C4—C4A—C5—N6	166.94 (14)	C11—C11A—C12—N13	−144.36 (13)
C13A—C4A—C5—N6	−12.7 (2)	C6B—C11A—C12—N13	−18.74 (16)
O1—C5—N6—C6A	174.40 (14)	O5—C12—N13—C13A	−3.1 (3)
C4A—C5—N6—C6A	−3.8 (2)	C11A—C12—N13—C13A	−179.33 (14)
O1—C5—N6—C14	13.7 (2)	O5—C12—N13—C6A	176.28 (15)
C4A—C5—N6—C14	−164.46 (13)	C11A—C12—N13—C6A	0.03 (17)
C5—N6—C6A—N13	29.51 (19)	N6—C6A—N13—C12	137.81 (13)
C14—N6—C6A—N13	−170.02 (13)	C6B—C6A—N13—C12	18.44 (16)
C5—N6—C6A—C6B	142.89 (15)	N6—C6A—N13—C13A	−42.78 (18)
C14—N6—C6A—C6B	−56.63 (17)	C6B—C6A—N13—C13A	−162.15 (13)
N13—C6A—C6B—C7	−150.21 (14)	C2—C1—C13A—C4A	−1.4 (2)
N6—C6A—C6B—C7	91.92 (17)	C2—C1—C13A—N13	−179.33 (15)
N13—C6A—C6B—C11A	−28.44 (15)	C4—C4A—C13A—C1	2.2 (2)
N6—C6A—C6B—C11A	−146.31 (13)	C5—C4A—C13A—C1	−178.11 (14)
C11A—C6B—C7—C7A	53.19 (16)	C4—C4A—C13A—N13	−179.72 (14)
C6A—C6B—C7—C7A	172.26 (13)	C5—C4A—C13A—N13	−0.1 (2)
C6B—C7—C7A—C10A	−20.2 (2)	C12—N13—C13A—C1	26.6 (2)
C6B—C7—C7A—O8	156.02 (14)	C6A—N13—C13A—C1	−152.68 (15)
C10A—C7A—O8—C9	0.07 (18)	C12—N13—C13A—C4A	−151.33 (15)
C7—C7A—O8—C9	−176.82 (14)	C6A—N13—C13A—C4A	29.3 (2)
C7A—O8—C9—C10	−0.28 (19)	C5—N6—C14—C15	74.64 (18)
O8—C9—C10—C10A	0.37 (19)	C6A—N6—C14—C15	−86.95 (16)
O8—C7A—C10A—C10	0.14 (18)	C18—O2—C15—C16	0.29 (19)
C7—C7A—C10A—C10	176.67 (16)	C18—O2—C15—C14	−175.39 (15)
O8—C7A—C10A—C11	−178.89 (14)	N6—C14—C15—C16	−105.4 (2)
C7—C7A—C10A—C11	−2.4 (3)	N6—C14—C15—O2	68.88 (18)
C9—C10—C10A—C7A	−0.31 (19)	O2—C15—C16—C17	0.04 (19)
C9—C10—C10A—C11	178.60 (16)	C14—C15—C16—C17	174.57 (18)
C7A—C10A—C11—C19	112.13 (17)	C15—C16—C17—C18	−0.4 (2)
C10—C10A—C11—C19	−66.6 (2)	C16—C17—C18—O2	0.6 (2)
C7A—C10A—C11—C11A	−9.5 (2)	C15—O2—C18—C17	−0.5 (2)
C10—C10A—C11—C11A	171.78 (16)	C10A—C11—C19—O3	−109.14 (18)
C10A—C11—C11A—C12	166.74 (13)	C11A—C11—C19—O3	9.5 (2)
C19—C11—C11A—C12	45.47 (18)	C10A—C11—C19—O4	69.78 (18)
C10A—C11—C11A—C6B	45.13 (17)	C11A—C11—C19—O4	−171.58 (14)

### Hydrogen-bond geometry (Å, °)

**Table:**

*D*—H···*A*	*D*—H	H···*A*	*D*···*A*	*D*—H···*A*
O4—H4O···O1^i^	0.93	1.75	2.671 (2)	174
C2—H2···O3^ii^	0.95	2.42	3.326 (2)	160
C3—H3···O3^iii^	0.95	2.56	3.384 (2)	146
C7—H7B···O4^iv^	0.99	2.54	3.455 (2)	155
C11A—H11A···O1^v^	1.00	2.38	3.325 (2)	157

Symmetry codes: (i) −*x*+1, −*y*+1, −*z*; (ii) *x*+1, *y*, *z*; (iii) −*x*+2, −*y*+1, −*z*; (iv) *x*+1/2, −*y*+3/2, *z*+1/2; (v) −*x*+3/2, *y*+1/2, −*z*+1/2.
